# Pharmacological Validation of ASIC1a as a Druggable Target for Neuroprotection in Cerebral Ischemia Using an Intravenously Available Small Molecule Inhibitor

**DOI:** 10.3389/fphar.2022.849498

**Published:** 2022-03-24

**Authors:** Xin Qi, Jian-Fei Lu, Zi-Yue Huang, Yi-Jun Liu, Lu-Bing Cai, Xin-Lan Wen, Xing-Lei Song, Jian Xiong, Pei-Yi Sun, Hao Zhang, Ting-Ting Zhang, Xuan Zhao, Qin Jiang, Ying Li, Oleg Krishtal, Leng-Chen Hou, Michael X. Zhu, Tian-Le Xu

**Affiliations:** ^1^ Center for Brain Science of Shanghai Children’s Medical Center, Shanghai Jiao Tong University School of Medicine, Shanghai, China; ^2^ Department of Anatomy and Physiology, Shanghai Jiao Tong University School of Medicine, Shanghai, China; ^3^ Department of Integrative Biology and Pharmacology, McGovern Medical School, The University of Texas Health Science Center at Houston, Houston, TX, United States; ^4^ Department of Dermatology, Xinhua Hospital, Shanghai Jiao Tong University School of Medicine, Shanghai, China; ^5^ Department of Anesthesiology, Shanghai 10th People’s Hospital, Tongji University School of Medicine, Shanghai, China; ^6^ Department of Cellular Membranology, Bogomoletz Institute of Physiology of NAS Ukraine, Kyiv, Ukraine

**Keywords:** ASIC1a, ischemic stroke, acidosis, neuroprotection, compound 5b

## Abstract

Acidosis is a hallmark of ischemic stroke and a promising neuroprotective target for preventing neuronal injury. Previously, genetic manipulations showed that blockade of acid-sensing ion channel 1a (ASIC1a)-mediated acidotoxicity could dramatically alleviate the volume of brain infarct and restore neurological function after cerebral ischemia. However, few pharmacological candidates have been identified to exhibit efficacy on ischemic stroke through inhibition of ASIC1a. In this work, we examined the ability of a toxin-inspired compound 5b (C5b), previously found to effectively inhibit ASIC1a *in vitro*, to exert protective effects in animal models of ischemic stroke *in vivo*. We found that C5b exerts significant neuroprotective effects not only in acid-induced neuronal death *in vitro* but also ischemic brain injury *in vivo*, suggesting that ASIC1a is a druggable target for therapeutic development. More importantly, C5b is able to cross the blood brain barrier and significantly reduce brain infarct volume when administered intravenously in the ischemic animal model, highlighting its systemic availability for therapies against neurodegeneration due to acidotoxicity. Together, our data demonstrate that C5b is a promising lead compound for neuroprotection through inhibiting ASIC1a, which warrants further translational studies.

## Introduction

Stroke is a common neurological disease, representing one of major causes of death and disability in the modern society (Collaborators et al., 2018). Cerebral ischemia occurs in about 87% of all strokes, leading to ischemic neuron death and consequently severe neurological deficits (Feigin et al., 2014). In addition to anoxia and energy failure, a series of damaging events related to death associated receptors also play important roles in neuronal injury (O’Collins et al., 2006). Cooperating with acute management of early revascularization by thrombolysis and mechanical thrombectomy, neuroprotective agents may extend the therapeutic window and prevent neuron death. In the past several decades, numerous studies have shown that glutamate-mediated excitotoxicity and the excessive activation of N-methyl-D-aspartate receptors (NMDARs), which represent the major ionotropic glutamate receptors responsible for excitotoxicity, significantly contribute to ischemic neuron death (Lipton, 2006). However, clinical studies targeting glutamate-related excitotoxicity have often failed to show efficacy in neuroprotection (Ikonomidou and Turski, 2002). Due to the essential and complex roles of glutamate in neurotransmission (Kalia et al., 2008), alternative molecular mechanisms involved in ischemic neuron death are also sought after and evaluated for their therapeutic potentials.

Acidosis caused by anaerobic respiration and metabolite accumulation is a hallmark of acute ischemic stroke and other chronic neurological diseases, including epilepsy, multiple sclerosis, traumatic brain injury, depression and headache (Wemmie et al., 2006). Brain imaging studies including MRI and PET found that ischemia-related pH decrease occurred immediately after occlusion and lasted for over 20 min after reperfusion, making it a sensitive biochemical marker of the ischemic core plus penumbra (Leigh et al., 2018). Since brain is an organ consuming huge amounts of oxygen and energy, reduced delivery of glucose during ischemia may cause ATP depletion and trigger fatal neuronal damage. However, attempts to improve energy supply by intravascular administration of glucose always exacerbate the brain injury both in rodents and humans (MacDougall and Muir, 2011; Bellolio et al., 2014). Hyperglycemia leads to a slight increase in ATP production but a severe drop of pH in penumbra, leading to worse outcome of ischemic stroke (Robbins and Swanson, 2014). It has been suggested that preventing acidosis-induced neuronal death may effectively alleviate the transformation of potentially reversible ischemic tissue into inevitable death core and thereby improve the neurological function (Leigh et al., 2018). Thus, searching for inhibitors that target the acidosis-related signaling pathway provides a new and promising strategy for the development of neuroprotective agents.

In the central nervous system, acid-sensing ion channels (ASICs) are the main receptors that detect the acidosis signal. Among them, the ASIC1a isoform forms proton-activated cation channels that account largely for the acidosis-induced neuronal injury in the cerebrum. Thus, the knockout of the *Asic1a* gene in mice protected the brain from ischemic injury, implicating ASIC1a as the main executor of acidotoxicity (Xiong et al., 2004). Our previous studies demonstrated that ASIC1a senses the pH drop caused by ischemia to contribute to neuronal death by intensifying calcium overload in a manner that depends on ASIC1a phosphorylation by Ca^2+^/calmodulin-dependent protein kinase II (CaMKII) (Gao et al., 2005). In addition, ASIC1a also aggravates cell death by interacting and thereby activating receptor-interacting serine/threonine-protein kinase 1 (RIPK1), a key component of necroptosis pathway, in a manner that is independent of the ion permeation but involves an acid-induced disruption of an intramolecular interaction between the N- and C-termini of the ASIC1a protein (Wang et al., 2015; Wang et al., 2020).

Pharmacologically, certain venoms from spider, snake, and sea anemone were found to significantly affect ASIC1a currents, including both activators and inhibitors (Cristofori-Armstrong and Rash, 2017). Based on X-ray crystallographic structural analysis, the acidic pocket and cysteine-rich thumb domain of ASIC1a are critical to the toxin-induced conformational changes and ion permeability of the functional channel (Baconguis and Gouaux, 2012; Baconguis et al., 2014; Sun et al., 2020). However, because of the poor stability and difficulty in delivery of the toxins *in vivo*, suitable pharmacological approaches remain scarce for effective ASIC1a inhibition in the brain. Since identification of lead compounds is both necessary and important for mechanism-based drug discovery (Santos et al., 2017), there is an urgent need to evaluate the neuroprotective effect of the currently available small molecule ASIC1a inhibitors systematically.

Previously, a novel competitive antagonist to ASIC1a was designed by using a molecular modeling approach based on the complexes containing ASIC1 and the highly selective and potent toxin-based ASIC1a inhibitor, Psalmotoxin 1 (PcTx1) (Buta et al., 2015). It was found that the PcTx1-inspired compound 5b (C5b) inhibited proton-evoked ASIC1a currents with an apparent IC_50_ of 27 nM at pH 6.7. Given these results, we asked whether C5b could be an effective compound targeting ASIC1a in the brain. In this work, we first confirmed the high selectivity and potency of C5b in blocking ASIC1a homotrimers or heterotrimers. We then showed that 10 μM C5b significantly prevented cell death induced by not only acidic exposure but also oxygen/glucose deprivation (OGD) of cultured primary cortical neurons. Since the ability to cross the blood-brain barrier (BBB) is the key to successful drug transformation, we examined the pharmacokinetics of C5b after intravenous administration and found that C5b rapidly dispersed into tissues and maintained a relatively constant concentration in the brain. Further experiments using a mouse model of cerebral ischemia, namely transient middle cerebral artery occlusion (MCAO), demonstrated a significant neuroprotective effect of C5b *in vivo* through either intracerebroventricular or intravenous route of administration. Taken together, our data show that by systemic delivery, C5b exerts a robust neuroprotective effect through inhibiting ASIC1a, providing a proof of concept for ASIC-targeting small molecule inhibitors to be developed as neuroprotective agents against acidotoxicity.

## Methods and Materials

### Animals

The experiments were performed on C57BL/6J, wild type (WT) and *Asic1a* knockout (KO, or *Asic1a* ^-/-^) mice. C57BL/6J mice were purchased from Shanghai Laboratory Animal Center, Chinese Academy of Sciences, Shanghai, China. The global *Asic1a* ^-/-^ mice (RRID: MGI_2654038) were the generous gifts of Prof. Michael J. Welsh (Howard Hughes Medical Institute, University of Iowa, Iowa City, IA) (Wemmie et al., 2002). The *in vivo* experiments were performed in 12-week-old male mice with a body weight of 22–25 g. All mice were bred in groups of 4 or 5 per cage under standard environment (12 h light/dark cycles at 21°C, 50%–60% humidity) of the specific pathogen-free class laboratory at Shanghai Jiao Tong University School of Medicine, with mouse chow and water *ad libitum*. Animal care and experimental protocols were approved by the Animal Ethics Committee of Shanghai Jiao Tong University School of Medicine, Shanghai, China (Policy Number DLAS-MP-ANIM.01–05). All efforts were made to minimize animal suffering and to reduce the number of animals used. For behavioral tests, animals were assigned in treatment groups in a random fashion and were habituated to the behavior testing room at least 1 h before the test.

### Primary Culture of Mouse Cortical Neurons and Cell Lines

Primary neurons were prepared from embryonic days 15–18 WT or *Asic1a* ^-/-^ mice and cultured as previous described (Song et al., 2021). Briefly, cerebral cortices were removed from the embryos, stripped of meninges and blood vessels under a dissecting microscope, and digested into dissociated cell with trypsin. Neurons were plated on poly-D-lysine-coated dishes in Neurobasal medium (Gibco, Cat. 21103049) with 1 mM glutamine and 2% B27 supplement. After 14 days in culture, the neurons were used to detect acidic or anoxic stimuli-evoked cell death.

Chinese hamster ovary (CHO) K1 cells were grown in Ham’s F12K medium (Gibco, Cat. 21127030) supplemented with 10% fetal bovine serum (FBS), 1% penicillin/streptomycin, and 1% Glutamax supplements at 37°C in a 5% CO_2_ humidified atmosphere.

### Anterior Cingulate Cortex Slice Preparation for Electrophysiological Recording

Acute coronal Anterior Cingulate Cortex (ACC) slices were prepared as previously described (Li et al., 2019). Briefly, mice were deeply anesthetized using 10% chloral hydrate with intubation and ventilation and then decapitated; brains were quickly removed and immersed in prechilled well-oxygenated (95% O_2_/5% CO_2_) artificial cerebrospinal fluid (aCSF) containing (in mM): 125 NaCl, 2.5 KCl, 12.5 D-glucose, 1 MgCl_2_, 2 CaCl_2_, 1.25 NaH_2_PO4, and 25 NaHCO_3_, pH 7.35–7.45. Forebrain was cut and glued onto the cutting slab and sectioned using a vibratome (Leica Microsystems VT 1000S) into 300-µm-thick coronal slices. For recovery, slices were incubated at 31°C in aCSF for at least 1 h. Then a piece of ACC containing slice was placed in a recording chamber and planked by a nylon mesh. The slice was observed with a microscope (Olympus, BX51WI) equipped with an infrared differential interference contrast video monitor. The recording bath temperature was maintained at 29–30°C using a heat circulator and exchanger for aCSF perfusion. Excitatory postsynaptic currents (EPSCs) were recorded from layer II/III pyramidal neurons and the stimulations were delivered at an intensity of 40 μA by a bipolar tungsten stimulating electrode placed in layer V/VI of the ACC.

### Whole-Cell Patch-Clamp Recordings

To test pH-dependence and selectivity of C5b inhibition, complementary DNA (cDNA) of *mAsic1a* (NM_009597.2), *mAsic2a* (NM_001034013.2) and *mAsic2b* (NM_007384.3) were constructed into pEGFP-C3 vector and transfected into CHO-K1 cell line by HilyMax liposome transfection reagent (Dojindo Laboratories, Cat. H357). For voltage clamp recording, cells were bathed in extracellular fluid (ECF) containing (in mM): 150 NaCl, 5 KCl, 2 CaCl_2_, 1 MgCl_2_, 10 glucose, and 10 HEPES, pH 6.0–7.4 adjusted with Tris-base. The pipette solution (internal) contained (in mM): 120 KCl, 30 NaCl, 1 MgCl_2_, 0.5 CaCl_2_, 5 EGTA, 2 MgATP, 0.3 NaGTP, and 10 HEPES, pH 7.4 adjusted with Tris-base. Cells were held at −60 mV in whole-cell mode and currents were digitized at 10 kHz and filtered at 2 kHz. All data were acquired in the voltage-clamp mode using Axon Digidata 1550B and MultiClamp 700B amplifier.

### Cell Death Assays

Cell death was assayed as previously described (Wang et al., 2020). For acidotoxicity, neurons were treated for 1 h with the pH 6.0 ECF in the absence and presence of C5b, which was then followed by incubation in the normal culture medium (fresh neurobasal medium supplemented with B27) for 24 h (reperfusion). For excitotoxicity, neurons were treated with 1 mM glutamate and 10 μM glycine in ECF for 1 h and then returned to the normal culture medium for 24 h. For OGD model, neurons were cultured in a glucose-free pH 7.4 ECF for 1 h at 37 °C with 1% O_2_ atmosphere and then returned to the normal culture medium for 24 h. Cell viability was assessed using the lactate dehydrogenase (LDH) assay kit following the manufacturer’s instructions (Promega, Cat. G1780). Briefly, an aliquot of the medium (100 μl) was transferred from the culture wells to the wells of a 96-well plate and mixed with 100 μl of the reaction solution provided by the kit. Optical density was measured at 492 nm 45 min later using the Synergy HTX multi-mode microplate reader (BioTek Instruments). Background absorbance at 620 nm was subtracted. For calcein and propidium iodide (PI) staining, cells were stained with 10 μM Calcein-AM (Dojindo Laboratories, Cat. C326) and 10 μg/ml PI (Dojindo Laboratories, Cat. P346) for 30 min at 37 °C and then washed for three times by phosphate-buffered saline. Calcein-positive or PI-positive cells were then examined by fluorescence microscopy and analyzed by ImageJ software. All death assays were performed with more than four biological repeats each time.

### Drug Delivery

For intracerebroventricular (ICV) microinjection, animals were anaesthetized and placed in a stereotaxic apparatus. A microliter syringe (Hamilton) was used to inject the drug to the lateral ventricle following the coordinates as below: anteroposterior, −0.5 mm; lateral, +1.5 mm; dorsoventral, −2.5 mm. After injection, the syringe was kept in the same position for 10 min before removal. Adult male wild type C57BL/6 mice (20–25 g) were randomly divided into two groups: saline treatment group (1 μL saline intracerebroventricularly injected at the rate of 0.1 μL/min) and C5b treatment group (1 μL C5b at 10 mM in saline intracerebroventricularly injected at the rate of 0.1 μL/min). For intravenous (IV) administration, 5 mg/kg C5b was dissolved in saline and injected into mice through the caudal vein.

### Transient MCAO

The experimental protocols (ethics protocol number: A2018-004) were approved by the Animal Care and Use Committee of Shanghai Jiao Tong University School of Medicine, Shanghai, China. Surgical procedures for MCAO were performed as described previously (Wang et al., 2015; Wang et al., 2020). Briefly, animals were anesthetized using 10% chloral hydrate with intubation and ventilation. Rectal and temporalis muscle temperature was maintained at 37 ± 0.5°C with a thermostatically controlled heating pad. Adult mice were fastened supine, and a midline incision was made in the neck. The right external carotid artery (ECA) and common carotid artery (CCA) were exposed. A vessel clip was placed on the CCA, and the ECA was isolated. The ECA was ligated by two nylon sutures (6-0 suture) and was cut between two sutures. The internal carotid artery (ICA) was exposed and another vessel clip was placed on it. An incision was made near the suture on the ECA, then a monofilament (RWD, Cat. 907-00012-01) was inserted into ECA, crossing the carotid sinus and reaching the clip on the ICA. Then the clip was removed, and the monofilament was inserted until the black symbol on the monofilament disappeared in the carotid sinus. The cerebral blood flow was stably reduced to below 20%, as monitored by transcranial laser Doppler (Moor Instruments Ltd.). After 1 h of occlusion, the monofilament and CCA vessel clip were removed, and the ECA was closed. The incision in the neck was sutured. After 24 h reperfusion, mice were tested for motor ability and then sacrificed for TTC (2, 3, 5-triphenyltetrazolium hydrochloride) staining.

### Rotarod Test

The rotarod test was performed to evaluate the motor coordination of the mice. Mice were placed on a rotating cylinder accelerating from four to forty rotations per minute. The latency to fall from the cylinder of each mouse was recorded in seconds and a comparison was made between the two groups. The data were collected and analyzed by an experimenter who was blind to the randomization of the experimental groups. All mice were trained three times per day for 3 days before testing.

### Grid-Walking Test

Grid-walking test was used to examine the motor coordination impairment after MCAO modeling. Mice were placed on a metal square grid with each grid cell being 1 × 1 cm^2^, and a camera was placed below the grid to record the movement of the mice. A “foot fault” is counted each time when the left front limb inaccurately slipped from the metal grid cell. The numbers of “foot faults” and precise stepping were recorded respectively until the sum of them reached 50. The motor coordination impairment was evaluated by the ratio: “foot faults”/50. The data were collected and then analyzed by an experimenter who was blind to the randomization of the experimental groups.

### TTC Staining

Mice were euthanized after 24 h of ischemia-reperfusion, and brains were sectioned coronally at 1-mm thickness and stained with 1% TTC (Sigma, Cat. T8877). After being incubated at 37 °C for 5 min at each side, the brain slices were fixed by 4% paraformaldehyde. ImageJ software was used to analyze the brain damage. The red and white areas represented the healthy and infarcted regions, respectively. The infarct volume was expressed as the percentage of the infarct volume compared with the total volume of the brain slices.

### High-Performance Liquid Chromatography-Mass Spectrometry

Plasma and brain samples were collected from five male mice at each time point and mixed with acetonitrile containing diazepam (50 ng/ml) as the internal standard at the room temperature. After centrifugation at 12,000 x g for 10 min, the supernatant of each sample was collected.

Samples were analyzed by LC-MS using an LCMS-8045 Mass Spectrometer (Shimadzu). HPLC separation was carried out on an LC-40D XR system (Shimadzu) using a Shim-pack column (100 × 2.1 mm, 1.9 µm particles diameter). Mobile phase A was 0.1% formic acid in water, and mobile phase B was acetonitrile. The flow rate was 0.3 ml/min and the elution gradients were programmed as following: initial, 20% B; 0.5 min, 75% B; 1.5 min, 75% B; 2.2 min, 20% B; 0.3 min, 20% B; 1.5 min, stop. The MS detector was equipped with the electrospray ionization and operated in the positive ion mode and MRM scan type. Operational electrospray ionization parameters were: spray voltage +3000 V, turbo ion spray temperature 300°C.

### Statistical Analysis

The pharmacokinetic calculations were commissioned by TopScience service which possessed a license of WinNonlin software. Other data were statistically analyzed by GraphPad Prism Software (version 8.4.3) using two-tailed Student’s t-test, paired t-test, or ANOVA as specified and presented as mean ± SEM. *p* < 0.05 was considered statistically significant.

## Results

### C5b is a Highly Selective Competitive Antagonist of ASIC1a

**FIGURE 1:**
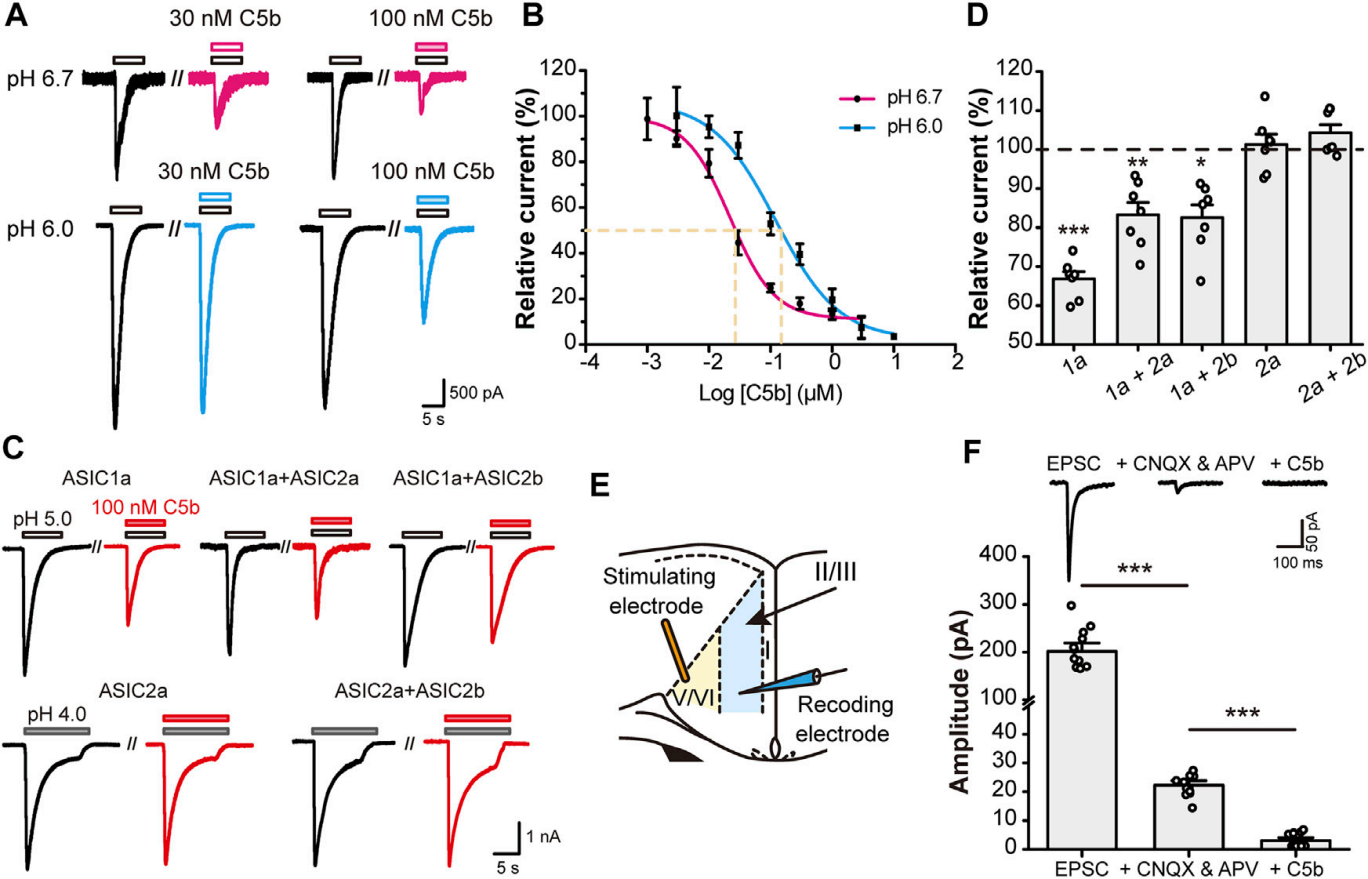
C5b specifically inhibited ASIC1a-mediated currents. **(A)** Representative traces of C5b inhibition of ASIC1a currents evoked at pH 6.7 (top row) and pH 6.0 (bottom row) at indicated concentrations. CHO-K1 cells expressing mASIC1a were bathed in a pH 7.4 extracellular fluid (ECF) before exposing to the pH 6.7 or pH 6.0 ECF. C5b was added with acidic stimulus as indicated. **(B)** pH dependence of C5b inhibition of ASIC1a. The acid-evoked currents were normalized to that in the absence of C5b. The data points (*n* = 5 for each) were fitted by the Hill equation, which yielded IC_50_ of 22.2 nM for pH 6.7 and IC_50_ of 121.9 nM for pH 6.0. **(C)** Representative traces of paired acid stimulations of ASIC currents in the absence (*black*) and presence (*red*) of 100 nM C5b. CHO-K1 cells were co-transfected with GFP and the indicated plasmids and used for whole-cell patch-clamp recording 24 h later. GFP-positive cells were first stimulated with the acidic ECF in the absence of C5b and then in the presence of 100 nM C5b at 3 min after the washout of the original acid-evoked current (I_0_). **(D)** Peak currents in the presence of 100 nM C5b normalized to the peak I_0_ for conditions shown in **(C)**. *n* = 7 for each group. Statistical significance for each group was determined by one sample t test compared with the hypothetical value of 100% for no inhibition. *p < 0.05, **p < 0.01, ***p < 0.001. **(E)** Schematic diagram of electrophysiological stimulation and recording of EPSCs in anterior cingulate cortex (ACC) in mouse brain slice. **(F)** C5b sensitivity of EPSCs of ACC pyramidal neurons. EPSCs were recorded as in **(E)** in the absence of any drug (*left*) or in the presence of 20 μM CNQX and 50 μM APV to block the glutamate-dependent postsynaptic currents (*middle*) or 20 μM CNQX, 50 μM APV, and 100 nM C5b to block the remaining currents mediated by ASIC1a. *n* = 11 for each group from four independent experiments. Statistical significance was determined with paired t test. ***p < 0.001. All data are mean ± SEM.

Since C5b binds to the acidic pocket of ASIC1a, we first examined whether the inhibition efficiency of C5b is dependent on extracellular pH. We exposed mouse (m) ASIC1a-transfected CHO cells to different concentrations of C5b while recording the whole-cell currents elicited by low pH, either pH 6.0 or pH 6.7. The dose-response curve of C5b exhibited a dramatic leftward shift under pH 6.7 activation of ASIC1a as compared to pH 6.0 (Figures 1A,B), with IC_50_ values being ∼22 nM at pH 6.7 and ∼100 nM at pH 6.0. This pH-dependent shift in apparent affinity of C5b is consistent with that described previously (Buta et al., 2015).

ASIC1a is the major subunit of the ASICs that mediate acidotoxicity during neuronal injury, which may act as homomeric ASIC1a or heteromeric ASIC1a-ASIC2 channels (Waldmann et al., 1997; García-Añoveros et al., 1997; Bartoi et al., 2014). In addition, homomeric ASIC2a and heteromeric ASIC2a-ASIC2b channels also exist in brain neurons (Price et al., 1996; Baron et al., 2002). To verify if C5b also inhibits the ASIC1a-ASIC2 heteromeric channels and ASIC2-based channels, we co-transfected mouse ASIC1a, ASIC2a, and ASIC2b in various combinations into CHO cells and measured the effect of C5b on proton-evoked currents (Figure 1C). Interestingly, while C5b (100 nM) inhibited the current elicited by a pH 5.0 solution in cells that expressed only ASIC1a by 35%, it suppressed that in cells co-expressing ASIC1a and ASIC2a or ASIC2b by only 20%. Moreover, C5b (100 nM) did not exhibit any inhibitory effect on currents elicited by a pH 4.0 solution in cells that expressed ASIC2a or ASIC2a plus ASIC2b (Figure 1D). Although these results do not rule out the possibility that C5b may also inhibit ASIC2 channels at less acidic conditions, the dependence of the ASIC2 channels on the extremely low pH makes them unlikely targets of C5b during tissue acidosis associated with cerebral ischemia, which has a pH range of 6.0–6.7 (Huang et al., 2015; Cui et al., 2016). These results indicate that C5b likely acts specifically at the ASIC1a-containing channels during acidotoxicity.

Previous studies showed that ASIC1a plays a key role in excitatory synaptic transmission (Du et al., 2014; Kreple et al., 2014; Yu et al., 2018; Li et al., 2019). To test whether C5b is able to inhibit the ASIC1a component of EPSCs, we prepared brain slices from mouse ACC and performed voltage-clamp recording (Figure 1E) as previously described (Li et al., 2019). The ASIC1a component of EPSCs was isolated by the addition of AMPAR (α-amino-3-hydroxy-5-methyl-4-isoxazole propionic acid receptor) and NMDAR blockers (20 μM CNQX and 50 μM APV) (Figure 1F). A further application of C5b (100 nM) completely abolished this small remaining component of EPSCs (Figure 1E), consistent with the notion that ASIC1a-containing channels account for the main part of the non-AMPAR/non-NMDAR-mediated EPSCs in cortical neurons. Taken together, these data indicate that C5b can selectively inhibit the current mediated by ASIC1a-containing channels in both heterologous systems and native neurons during synaptic transmission.

### C5b Alleviates Neuronal Cell Death in an ASIC1a-Dependent Manner

**FIGURE 2:**
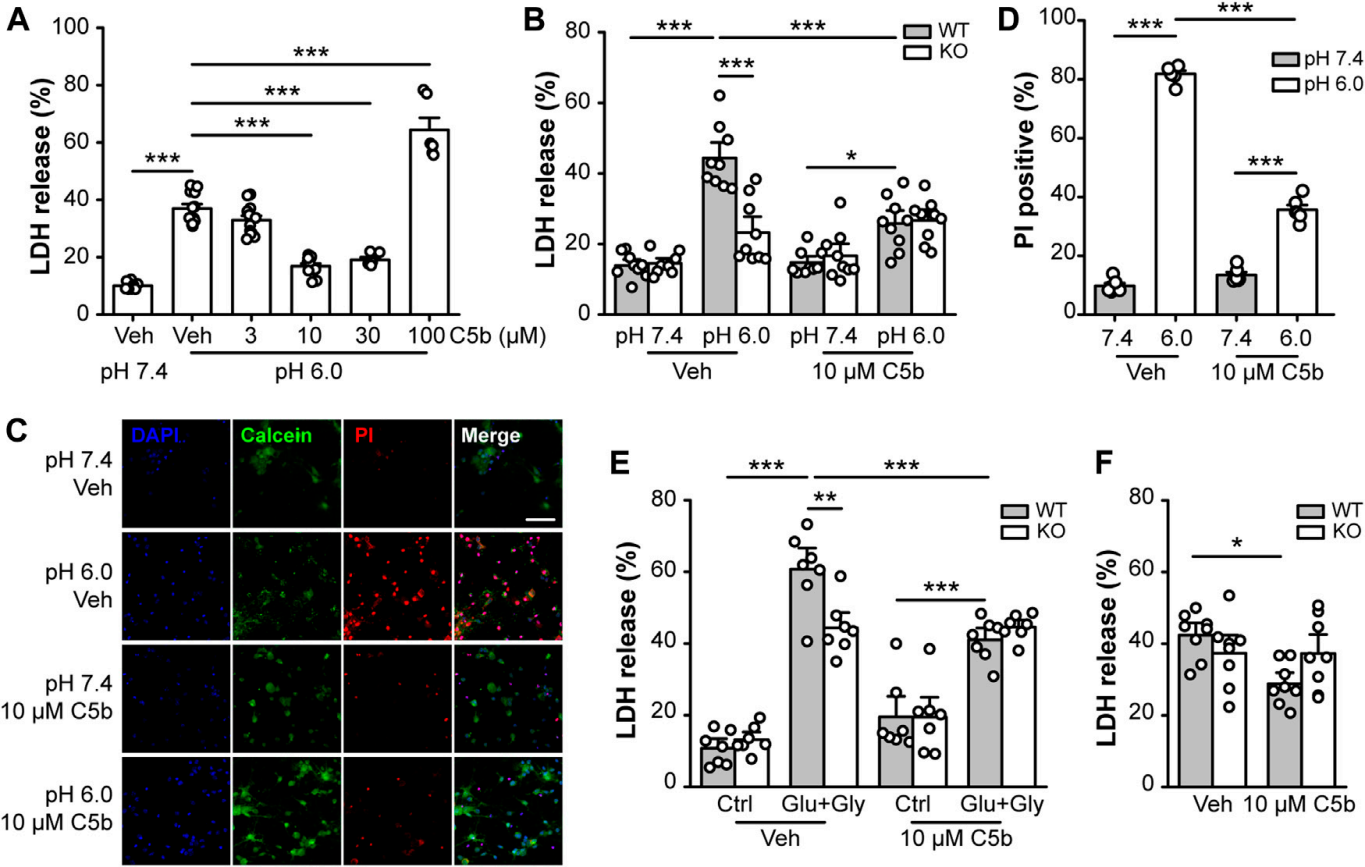
C5b protected neurons from ASIC1a-dependent cell death. **(A)** Concentration dependence on C5b of the protective effect against acid (pH 6.0)-induced neuronal death as detected by the LDH release assay. Mouse cortical neurons were treated with a pH 6.0 ECF for 1 h and then returned to the normal culture medium for 24 h C5b was present during the acid treatment but was omitted during the recovery period. *n* = 5 or 9 for each group. Statistical significance was determined with one-way ANOVA for differences across groups at different doses in conjunction with Tukey test for means comparison. ***p < 0.001. **(B)** C5b (10 µM) inhibited acid (pH 6.0)-induced death of WT primary cortical neurons, but had no effect on *Asic1a* KO neurons. *n* = 9 for each group. **(C)** Representative images of mouse cortical neurons treated with pH 7.4 and pH 6.0 ECF, with cell death detected by Calcein (*green*) and PI (*red*) staining. DAPI (*blue*) staining was used for cell counting. Neurons were treated with vehicle (veh) or C5b (10 µM). Scale bar, 20 μm. **(D)** Statistics for **(C)** showing that 10 μM C5b dramatically reduced PI-positive cells under the condition of 1 h acidosis followed by 24 h reperfusion. *n* = 6 for each group. **(E)** C5b (10 µM) suppressed glutamate-induced neuronal death in WT neurons but had no effect on *Asic1a* KO neurons. Excitotoxic death was induced by a treatment of 10 μM glycine and 1 mM glutamate for 1 h followed by returning to the normal culture medium for 24 h. *n* = 7 for each group. **(F)** C5b (10 µM) attenuated neuronal death induced by OGD in WT neurons but had no effect on *Asic1a* KO neurons. Cell death was induced by OGD for 1 h followed by reoxygenation for 24 h. *n* = 8 for each group. All data are pooled from two to four independent experiments and are shown as mean ± SEM. In **(B–F)**, statistical analysis was performed by two-way ANOVA followed by the Tukey test to identify significant treatment and genotype factors and their interactions. *p < 0.05, **p < 0.01, ***p < 0.001.

Next, we examined whether C5b can rescue neurons from acid-induced cell death in primary cultured mouse cortical neurons. The cortical neurons were exposed to an extracellular solution of pH 7.4 (control) or pH 6.0 (acid treatment) for 1 h followed by recovery in the normal culture medium for 24 h. Cell death was assessed using the LDH assay and expressed as the fraction of LDH detected in the culture medium versus the total LDH from lysed cells. The acid treatment increased the LDH release from ∼10% in pH 7.4-treated neurons to ∼40% in the pH 6.0-treated neurons (Figure 2A). C5b, at 10 and 30 μM, significantly suppressed the pH 6.0-induced LDH release; however, at 100 μM, it enhanced LDH release (Figure 2A), indicating a protective effect of C5b against acid-induced neuronal death at the moderate concentrations, but cytotoxicity of C5b at high concentrations. To confirm that the protective effect of C5b occurred through its inhibition of ASIC1a, we compared the effect of C5b on acid-induced cell death in WT and *Asic1a* KO neurons. As shown in Figure 2B, while C5b (10 µM) markedly reduced the increase in LDH release from WT neurons induced by the pH 6.0 solution, it failed to exert any detectable effect on *Asic1a* KO neurons treated with the acidic solution. As an alternative method to assess cell death, we also used PI staining, a DNA-binding dye that labels dead cells due to destruction of membrane integrity. In vehicle-treated WT cortical neurons, the exposure to the pH 6.0 solution dramatically increased the fraction of PI-positive cells from ∼10% at pH 7.4 to >80%, but with the addition of C5b (10 µM), this acid-induced increase was greatly reduced to only 35.6 ± 1.6% (Figures 2C,D), further demonstrating that C5b can alleviate the acid-induced cell death.

Previously, we showed that in addition to acidotoxicity, ASIC1a also contributes to excitotoxicity by amplifying the NMDAR-mediated neuronal damage due to phosphorylation by CaMKII (Gao et al., 2005). To test whether C5b also exerts a protective effect on this form of ASIC1a-mediated neuronal death, we performed LDH assay on primary cortical neurons subjected to NMDAR excitotoxicity with the treatment of 1 mM glutamate (Glu) and 10 μM glycine (Gly) for 1 h. Consistently, we found that the Glu+Gly treatment induced a greater increase in LDH release in WT neurons than in *Asic1a* KO neurons, demonstrating the presence and proportion of the ASIC1a-dependent component in the NMDAR excitotoxicity (Figure 2E). The addition of C5b (10 μM) specifically reduced the NMDAR excitotoxicity to WT neurons to the level similar to that achieved in the *Asic1a* KO neurons (Figure 2E). The lack of effect of C5b on *Asic1a* KO neurons in this model further confirms that C5b exerts its neuroprotective effect through acting at ASIC1a. Furthermore, we subjected the primary cortical neurons to OGD (1 h) followed by re-oxygenation (24 h) to mimic ischemia/reperfusion under *in vivo* pathological conditions. Again, C5b (10 µM) significantly attenuated the OGD-induced death of WT neurons, but had no effect on *Asic1a* KO neurons (Figure 2F). Taken together, these data indicate that C5b exerts neuroprotective effects by alleviating ASIC1a-mediated neuronal death.

### C5b is a Blood-Brain Barrier Permeable Agent

Given the importance of ASIC1a in brain injury associated with ischemic stroke, it would be of interest to know if C5b also exerts neuronal protective effect *in vivo* under this pathological condition. However, one of the major obstacles of developing central nervous system (CNS)-acting drugs is the BBB. BBB plays a very important role in maintaining CNS homeostasis, but it also hurdles brain drug delivery, as it blocks almost 100% of large molecule drugs and more than 98% of small molecule drugs from their active targets in the CNS (Gabathuler, 2010). Therefore, we first determined whether C5b as a lead compound can penetrate the BBB and achieve sufficient exposure in the CNS through peripheral administration.

**FIGURE 3:**
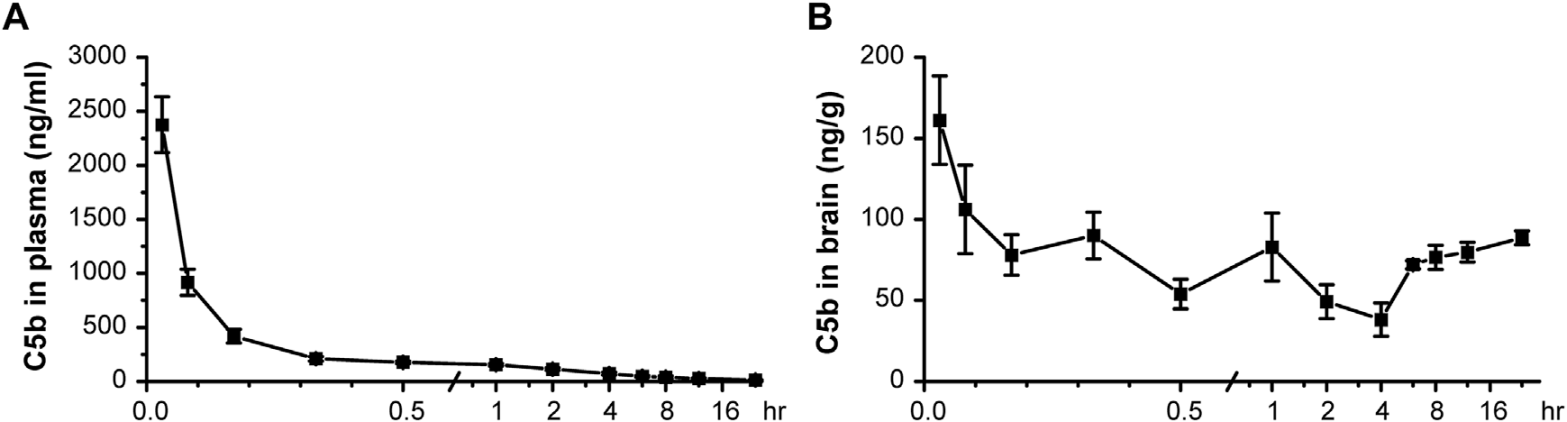
Pharmacokinetic test *in vivo* indicated that C5b is BBB permeable. **(A)** C5b (5 mg/kg dissolved in saline) was injected into mice through the caudal vein. The plasma concentrations of C5b at different times after the intravenous administration were accessed from whole blood and detected by HPLC-MS/MS. *n* = 5 for each point. **(B)** Whole brains were dissected at different times after the intravenous administration and homogenized for detection of the C5b concentration by HPLC-MS/MS. *n* = 5 for each point. Concentrations were calculated based on the standard curve prepared in advance. Data are mean ± SEM.

Based on an analysis of its physicochemical properties, we found C5b to basically possess all main features of a standard BBB-permeable agent (Supplementary Table S1), as indicated by data from PubChem (Kim et al., 2021). Next, we administered C5b (5 mg/kg) through intravenous (IV) injection into mouse caudal vein and then collected blood and brain tissues at various time points after the injection. The concentrations of C5b in blood and brain tissues were determined by high performance liquid chromatography-tandem mass spectrometry (HPLC-MS/MS) with a retention time of 3.35 min. We found that the concentration of C5b in plasma dropped dramatically within the first half hour, indicating that C5b exhibits a fast tissue distribution (Figure 3A). To calculate the primary pharmacokinetic parameters of C5b, we used the statistical moment method of the WinNonlin software (Supplementary Table S2). The ∼6.5 h elimination half-life (t_1/2_z) and the ∼640 h μg/L area under the curve (AUC_0-t_) values indicate that C5b exhibits a wide and fast tissue distribution, and is not rapidly metabolized. These results suggest that intravenously administered C5b may have enough time to cross the BBB and spread throughout the brain to interact with its CNS targets.

Indeed, we detected C5b in brain tissues as early as 2 min after the IV administration at the dose of 5 mg/kg, which remained relatively stable during the 24 h sampling time period (Figure 3B). These data demonstrate that C5b can pass through the BBB and reach a stable drug concentration in the brain for a long time to exert a pharmacological effect. This property of C5b prompted us to test if IV administration of this compound has a neuroprotective outcome in a stroke model.

### Intravenous Administration of C5b Attenuated Ischemic Cerebral Injury

**FIGURE 4:**
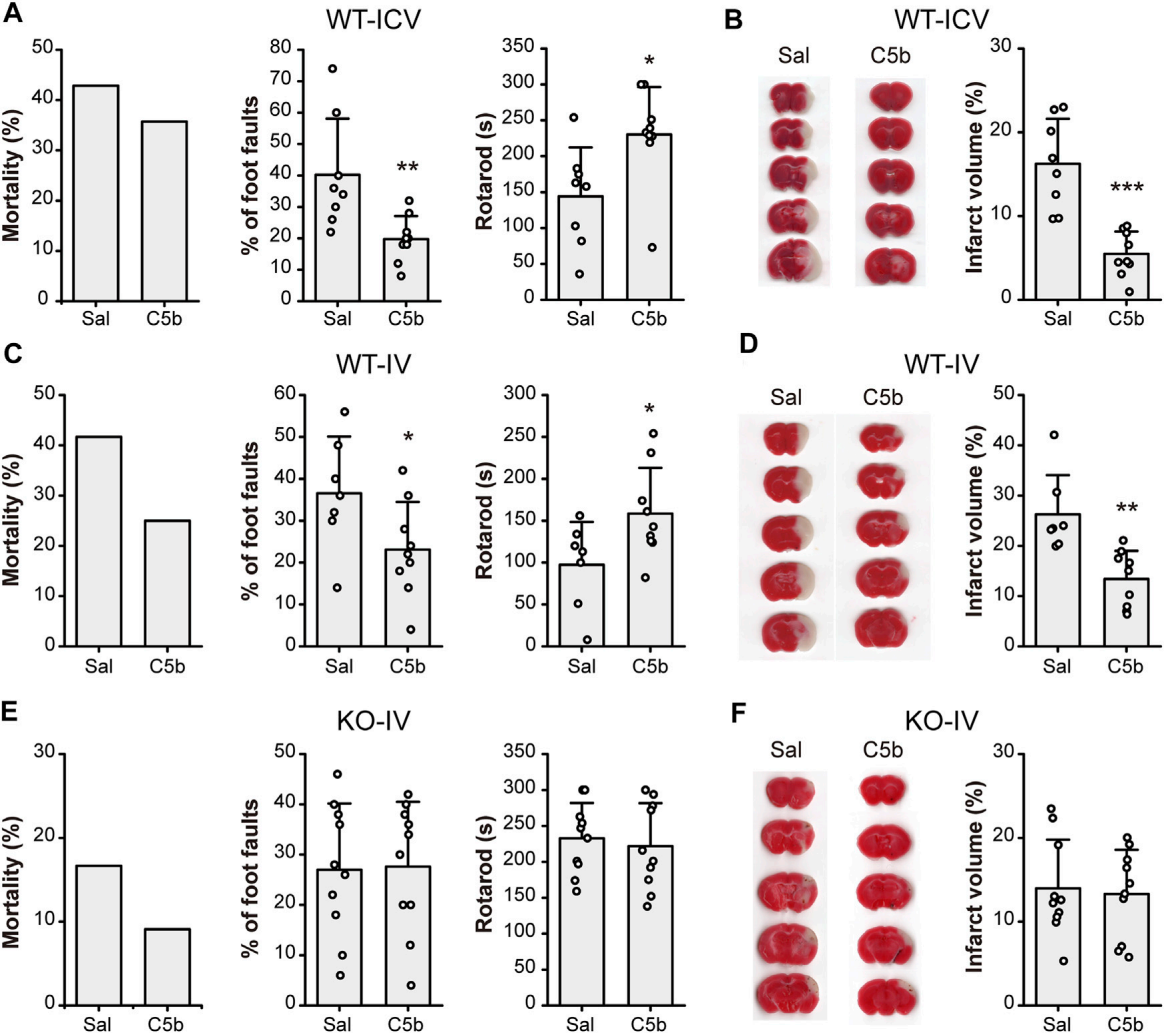
C5b attenuated neurological impairment and alleviated infarct volume in mouse MCAO model of ischemic stroke. **(A)** Mortality rate (*left*) and behavioral tests for motor ability (*middle*, grid-walking test; *right*, rotarod test) of WT mice with intracerebroventricular (ICV) saline or C5b (1 μL of 10 mM) treatment and subject to MCAO. *n* = 14 for each group. **(B)** TTC-staining of brain slices (*left*) and summary data of the infarct volume (*right*) of mice with ICV saline or C5b treatment. White areas stand for injured brain area while red represents uninjured areas. *n* = 8 or 9 for each group. **(C)** Mortality rate (*left*) and behavioral tests for motor ability (*middle*, grid-walking test; *right*, rotarod test) of WT mice with intravenous (IV) saline or C5b (5 mg/kg) treatment and subject to MCAO. *n* = 12 for each group. **(D)** TTC-staining of brain slices (*left*) and summary data of the infarct volume (*right*) of mice with IV saline or C5b treatment. *n* = 8 or 9 for each group. **(E)** Mortality rate (*left*) and behavioral tests for motor ability (*middle*, grid-walking test; *right*, rotarod test) of *Asic1a* KO mice with intravenous treatment and MCAO as in **(C)**. *n* = 11 for each group. **(F)** TTC-staining of brain slices (*left*) and summary data of the infarct volume (*right*) of KO mice with IV saline or C5b treatment. *n* = 10 for each group. All data are mean ± SEM. Statistical significance was determined with Student’s t test. *p < 0.05, **p < 0.01, ***p < 0.001.

To examine whether C5b has any neuroprotective effect *in vivo*, we first delivered C5b directly into the CNS through intracerebroventricular (ICV) injection into the lateral ventricle. Transient MCAO was used to introduce acute cerebral ischemia in mice, which included occlusion for 1 h followed by 24 h reperfusion. Then the mice were subjected to behavioral tests for their neurological functions and brain sections were stained with TTC for analysis of infarct size. Although the ICV administration of C5b (10 mM, 1 µL) 15 min before the MCAO only slightly decreased the mortality rate, it significantly improved the performance in foot-fault test and motor coordination in the rotarod test (Figure 4A), suggesting rescued neurological function after the MCAO as compared to control mice that were treated with the vehicle (saline). Moreover, the ICV administration of C5b effectively reduced the infarct volume as compared to the saline control (Figure 4B), suggesting that the compound protected neurons from death induced by cerebral ischemia.

Since C5b is BBB permeable, we next tested whether C5b is also neuroprotective against ischemic stroke when administered through IV injection, which is more practical for clinical usage than ICV injection. C5b (5 mg/kg) was injected intravenously 15 min before MCAO. Similar to the ICV delivery, the IV administration of C5b also decreased the mortality rate, with potentially an even better effect than ICV (Figure 4C). It also prominently attenuated cerebral injury including both the neurological function (Figure 4C) and the infarct volumes (Figure 4D) in an ASIC1a-dependent way, since no protection was observed in *Asic1a* KO mice with the C5b treatment (Figures 4E,F). Together, these data demonstrate that C5b is a promising candidate for developing neuroprotection drugs with CNS availability under ischemic conditions, where ASIC1a plays a significant role in neuronal injury.

## Discussion

Although stroke-related disorder has been one of the major causes of mortality and disabilities for decades, efforts on the development of neuroprotective agents based on the mechanism of cell death have yielded few effective drug candidates. Approaches targeting excitotoxity, which aims to stop the widespread activation of glutamatergic pathways and block calcium influx through NMDARs, have the promise in mitigating ischemic neuronal death; however, they also tend to hinder the recovery process after stroke (Lai et al., 2011). Some NMDAR antagonists, such as ketamine, MK-801, and gavestinel, have shown significant protective effects in animal stroke models, but none of them were approved for clinical use due to severe side effects. In this context, neuronal injury resulting from acidotoxicity, due to the frequent and severe acidosis that occur under stroke conditions, should be targeted either as an alternative treatment option or an additional therapy to complement the anti-excitotoxicity approach. Previous studies by us and other groups have demonstrated the pivotal role of ASIC1a, the major acid sensor in the CNS, in mediating ischemic cell death through multiple mechanisms, including the ion conduction-dependent crosstalk with the NMDARs (Gao et al., 2005; Duan et al., 2011) and the ion conduction-independent conformational signaling leading to neuronal necroptosis (Wang et al., 2015; Wang et al., 2020). The growing appreciation of the importance of acidosis in neuronal injury raises the intriguing possibility that targeting the ASIC1a-dependent acidotoxicity may provide an alternative but promising approach for developing novel neuroprotective agents to aid the clinical management of stroke. However, although some pharmacological agents have been found to inhibit the ASIC1a-containing channels with high potency and high selectivity (Wemmie et al., 2013; Baron and Lingueglia, 2015), such as PcTx1 (Escoubas et al., 2000) and mambalgin-1 (Diochot et al., 2012), these toxin-derived ASIC inhibitors, unfortunately, are unlikely to be used in the clinics because of their poor stability and difficulty in delivery. Therefore, there is an urgent need to develop novel small molecule ASIC1a inhibitors and to evaluate their neuroprotective properties.

In this study, we demonstrate that a recently-identified novel ASIC1a inhibitor, C5b, which binds to the acidic pocket of ASIC1a (Buta et al., 2015), can potently and selectively inhibit acid-induced activation of ASIC1a-containing channels, including both ASIC1a homotrimers and ASIC1a-ASIC2 heterotrimers, in a pH-dependent manner. More interestingly, C5b is able to attenuate not only the acid-induced cell death, but also the death of primary cultured cortical neurons induced by glutamate-evoked excitotoxity or OGD. Importantly, C5b did not exert a protective effect in *Asic1a* KO neurons subject to any of the treatments, demonstrating the specificity of this compound on the ASIC1a-dependent component in these death pathways. Moreover, these results also corroborate the notion that ASIC1a contributes significantly to glutamate-stimulated excitotoxicity *via* NMDARs as well as the death induced by OGD/reoxygenation. Therefore, the *in vitro* findings here support the exciting possibility that C5b may be neuroprotective under conditions of cerebral ischemia, in which ischemia/reperfusion trigger both excitotoxicity and acidotoxicity. Importantly, C5b shows a sharp decrease in plasma concentration with systemic administration, suggesting that the pharmacokinetic characteristics of C5b favor rapid tissue distribution, conferring it as a good candidate for *in vivo* use in behaving animals. As expected, we detected a marked neuroprotective effect of C5b in a mouse MCAO model of ischemic stroke. Both neurological dysfunction and infarct volume were greatly blunted by the administration of C5b as compared to the vehicle control. Of particular note, the neuroprotection against ischemic brain injury by C5b was achieved by not only ICV injection but also IV administration, a clinically relevant route of drug delivery. These results indicate a promising translation potential of C5b in treating cerebral ischemia in the future.

Compared with previously used channel blockers, such as amiloride which shows poor selectivity on sodium channels or PcTx1 which has difficulty in drug delivery, the toxin-inspired compound has a clear advantage in clinical practice. Functioning as a competitive inhibitor, the inhibitory efficacy of C5b significantly increased under mild acidosis, making it likely to be more effective in the penumbra region when compared to the ischemic core. Meanwhile, the unique BBB-permeability of C5b should facilitate the accessibility of drug administration *in vivo*, which should pave the way for future evaluation of the pharmacologic effects of the drug treatment at different ischemic periods during and/or after occlusion and reperfusion. Overall, C5b is a promising lead compound specifically targeting ASIC1a and yielding effective neuroprotection under neurotoxic conditions both *in vitro* and *in vivo*. Future work should aim to further optimize C5b for the purpose of developing new neuroprotective agents with improved stability and sensitivity to treat acidosis-related cerebral diseases.

## Data Availability

The raw data supporting the conclusion of this article will be made available by the authors, without undue reservation.
