# Supplementary material for: Pharmacological Validation of ASIC1a as a Druggable Target for Neuroprotection in Cerebral Ischemia Using an Intravenously Available Small Molecule Inhibitor
Source: Front Pharmacol. 2022 Mar 24;13:849498. doi: 10.3389/fphar.2022.849498 (PMC8988055; doi:10.3389/fphar.2022.849498)
Supplement: Supplementary file 1 [file DataSheet1.docx]

Title page

**Pharmacological validation of ASIC1a as a druggable target for neuroprotection in cerebral ischemia using an intravenously available small molecule inhibitor**

**Xin Qi^1,2^, Zi-Yue Huang^2†^, Yi-Jun Liu^2†^, Lu-Bing Cai^2†^, Xin-Lan Wen^2^, Xing-Lei Song^1^, Jian Xiong^3^, Pei-Yi Sun^4^, Hao Zhang^5^, Qin Jiang^1^, Ying Li^1^, Oleg Krishtal^6^, Leng-Chen Hou^5^, Jian-Fei Lu^2^, Michael Xi Zhu^3*^, Tian-Le Xu^1, 2*^**

^1^Center for Brain Science of Shanghai Children's Medical Center, Shanghai Jiao Tong University School of Medicine, Shanghai 200127, China.

^2^Department of Anatomy and Physiology, Shanghai Jiao Tong University School of Medicine, Shanghai 200025, China.

^3^Department of Integrative Biology and Pharmacology, McGovern Medical School, The University of Texas Health Science Center at Houston, Houston, TX 77030, USA

^4^Department of Dermatology, Xinhua Hospital, Shanghai Jiao Tong University School of Medicine, Shanghai 200092, China.

^5^Department of Anesthesiology, Shanghai 10th People's Hospital, Tongji University School of Medicine, Shanghai 200072, China.

^6^Department of Cellular Membranology, Bogomoletz Institute of Physiology of NAS Ukraine, Kyiv 01024, Ukraine

^†^These authors have contributed equally to this work and share senior authorship.

^*^Co-corresponding authors.

Email: xu-happiness@shsmu.edu.cn (T.-L.X.) and michael.x.zhu@uth.tmc.edu (M.X.Z.)

Leading contact: xu-happiness@shsmu.edu.cn (T.-L.X.)

**Supplementary Table 1. Chemical and physical properties of C5b**

| **Property Name** | **Property Value** | **Standard** |
| --- | --- | --- |
| PubChem CID | 122177620 | - |
| Molecular Weight | 426.9 Da | < 450 Da |
| XLogP3-AA | 4.7 | 2~5 |
| Hydrogen Bond Donor Count | 2 | < 3 |
| Hydrogen Bond Acceptor Count | 5 | < 10 |
| Rotatable Bond Count | 9 | < 10 |
| Topological Polar Surface Area | 82.6 Å² | < 90 Å² |

XLogP3-AA represented water partition coefficient computed by XLogP3 3.0. Other properties were all computed by Cactvs 3.4.8.18.

**Supplementary Table 2. Pharmacokinetic characteristics of C5b**

| **Parameter** | **Units** | **i.v. 5 mg/kg** |
| --- | --- | --- |
| AUC_(0-t)_ | hr•μg/L | 639.27±117.88 |
| AUC_(0-∞)_ | hr•μg/L | 702.06±118.92 |
| t_1/2_z | hr | 6.49±2.82 |
| Vz | L/kg | 62.90±18.23 |
| CLz | L/hr/kg | 7.28±1.17 |
| MRT _(0-∞)_ | hr | 6.57±2.64 |

AUC: area under the curve of C5b concentration in plasma; t_1/2_: terminal elimination half-life; V: apparent volume of distribution; CL: clearance rate; MRT: mean residence time.
